# Culturally adapted exercise program for postmenopausal women with excess weight gain: A modified international/local Delphi study

**DOI:** 10.1371/journal.pgph.0005083

**Published:** 2025-09-11

**Authors:** Isaac Mensah Bonsu, Corlia Brandt, Adedayo Tunde Ajidahun, Hellen Myezwa

**Affiliations:** 1 Department of Physiotherapy, School of Therapeutic Sciences, University of The Witwatersrand, Johannesburg, South Africa; 2 Faculty of Allied Health Sciences, Department of Physiotherapy and Sports Science, College of Health Sciences, Kwame Nkrumah University of Science and Technology, Kumasi, Ghana; University of Global Health Equity, RWANDA

## Abstract

Previous reports suggest that exercise intervention can reduce body weight and body composition profile parameters in obese postmenopausal women (PMW). Although some exercises have been constructed, international and local consensus on exercise programs for weight loss among postmenopausal women with excess weight (overweight and obesity) gain is lacking. We aimed to validate a culturally adapted exercise-based weight loss program for postmenopausal women using the Delphi consensus methodology. The study process consisted of three Delphi rounds among twelve international and twenty-one local experts. Participants were recruited via purposive sampling representing physiotherapists, exercise physiologists, exercise scientists, human kinetics and health education based on their interests and knowledge. A preliminary program was developed based on literature reviews and project findings. In the first Delphi round, open-ended responses were analysed thematically and converted into statements. Experts rated their agreement with these statements on a 5-point Likert scale in the second round. Consensus was defined as at least 60% strong agreement/disagreement. In the final round, participants reviewed the program and achieved an 80% consensus threshold. Thirty-three experts completed all three rounds, reaching consensus across four domains: health assessment, pre-exercise parameters, exercise dosage, and physician referrals. Response rates were high, with a final participation rate of 78% (n = 32).The high response rate established a strong consensus, providing clinicians with evidence-based recommendations to improve health outcomes for postmenopausal women in Ghana, with particular emphasis on the cultural adaptation process, especially the innovative inclusion of Ampe in the intervention.

## Introduction

Excess weight gain in postmenopausal women is a significant public health concern, associated with increased risks of cardiovascular diseases, diabetes, and metabolic disorders [1]. Weight gain during this period is influenced by hormonal changes, lifestyle factors, and the physiological adaptations of menopause, which affect metabolism and fat distribution [1–4]. Postmenopausal women face unique health challenges that require targeted interventions to improve their health and overall well-being [1]. Physical activity has been well-documented as essential in managing weight and reducing the risks of related health conditions [5–7]. However, the effectiveness of exercise programs can be influenced by sociocultural perceptions and practices, particularly in African contexts [8]. Therefore, culturally adapted exercise interventions are essential to addressing the specific needs of postmenopausal women.

Global evidence supports the role of structured exercise programs in weight management for postmenopausal women, with benefits ranging from improved cardiovascular health to enhanced muscle strength and flexibility [9,10]. However, generic, one-size-fits-all exercise programs often fail to consider different populations’ cultural, social, and environmental contexts [8]. In many low- and middle-income countries, including those in Africa, there are barriers to participation in structured physical activity programs, such as limited access to facilities, lack of culturally relevant exercise options, and low awareness of the importance of exercise for health promotion among women in post-menopause [11]. Thus, culturally adapted exercise interventions, tailored to the local needs and preferences of postmenopausal women, are essential for ensuring participation, adherence, and success.

Culturally adapted programs consider the social, cultural, and environmental contexts of participants, making the exercises more relevant, acceptable, and sustainable [12]. For postmenopausal women, this could involve designing programs that align with their cultural practices, daily routines, and preferred types of physical activity. Importantly, these adaptations can promote better adherence and long-term participation, as the exercises are more relatable and feasible within their lifestyle [12]. Therefore, this study aims to develop a culturally adapted exercise program for postmenopausal women with excess weight gain, using the modified Delphi technique, which integrates both international evidence and local expert consensus.

The Delphi method is particularly valuable for developing consensus on best practices or guidelines when evidence is incomplete or expert opinion is necessary [13]. This study employed a modified Delphi approach, incorporating international experts in women’s health and local health practitioners familiar with the cultural and contextual factors affecting postmenopausal women. By combining global expertise with local knowledge, we aim to create an exercise program that not only adheres to evidence-based guidelines but is also culturally sensitive and practical for the target population.

The objective of this study is to develop and validate a culturally adapted exercise program specifically designed for postmenopausal women with excess weight gain. Using a modified Delphi study, the research draws on international best practices and local expertise to create a program that promotes long-term engagement and improves health outcomes for postmenopausal women.

## Materials and methods

### Ethics statement

Ethical approval for this study was granted by the Human Research Ethics Committee of the University of the Witwatersrand, South Africa (Reference No. M190467). In Ghana, approval was obtained from the Committee on Human Research, Publication, and Ethics at Kwame Nkrumah University of Science and Technology, Kumasi (Reference No. 596/19, 621/21). Participants were informed that their participation was voluntary and that they reserve the right to withdraw from the study without any prejudice. Those who consented signed the consent forms and were guaranteed their anonymity in future reports and publications. Participants were informed that their contribution would help validate exercise-based programs to manage postmenopausal women with excess weight.

### Delphi Technique

#### Defining consensus.

The purpose of the Delphi technique is to arrive at a consensus on an appropriate culturally exercise-based programme for weight loss in Ghanaian postmenopausal women with excess body weight. For this study, 80% agreement was chosen as the threshold for consensus. This threshold was determined after a review of the literature, which indicated that consensus levels in Delphi studies typically range from 60% to 90%, depending on the context and objectives [14,15]. To support strong conclusions regarding the development of a weight loss program for postmenopausal women, a higher level of agreement was deemed necessary. This population presents with unique physiological, psychosocial, and cultural considerations that require careful attention in program design [1–4,12]. Given the potential health risks and variability in individual needs during the menopausal stage, it was essential to ensure that the proposed intervention strategies were widely accepted and considered relevant by the expert panel [2,14]. Setting the consensus threshold at 80%, a level supported in the literature as appropriate for generating reliable and actionable recommendations, helped ensure that only those statements reflecting a strong level of expert agreement were included in the final program. This threshold was established a priori, before data analysis commenced, to maintain methodological rigor and reduce potential bias.

### Study design

#### Panel selection.

The Delphi rounds were conducted between April and August 2022. The panel of experts was identified by the principal investigator through a systematic process involving professional boards, academic directories, and recommendations from senior colleagues within the relevant disciplines. A total of 102 invitations were sent via email to potential participants. Each invitation included a request for participation along with detailed information about the study. Three individuals declined participation due to being on sabbatical leave, while two others expressed initial interest but later withdrew due to time constraints. Ultimately, forty-one experts who met the predefined selection criteria provided informed consent and participated in the study.

#### Selection of participants.

The Delphi rounds were conducted from April to August 2022. Expertise was defined in the areas of “exercise program development” and exercise physiology. As a result, the principal researcher (IMB) sent invitations via email to 102 identified experts who met the eligibility criteria. The email included the study’s informed consent form and background information. Interested panellists were encouraged to contact the primary researcher (IMB) and share the study details with others who might be interested and meet the criteria. Three potential participants declined due to sabbatical leave, while two expressed interest but could not participate due to scheduling conflicts. Ultimately, 41 experts who met the selection criteria provided informed consent to participate. These experts, purposively selected, included physiotherapists, exercise physiologists, exercise scientists, and specialists in human kinetics and health education.

#### Development and validation of the questionnaire.

The outcomes of three prior investigations and the findings of the literature review were used to generate a list of questions for the first round of the Delphi survey. The preliminary result from the overall project established a high prevalence of excess weight gain among postmenopausal women using BMI, WHtR and WHR. The second study revealed that postmenopausal women do not engage in adequate physical activity, have poor lifestyles due to their eating patterns and believe that cultural backgrounds encourage them to gain weight. This study contributed to understanding their physical activity levels, lifestyle and cultural influences on weight gain. These results confirmed the need to incorporate culturally appropriate physical activity in the development of the exercise program. “Ampe”, which is a Ghanaian household indigenous recreational activity, was identified in the literature [16]. This physical activity (Ampe) involves clapping while singing and jumping vertically with two contestants at a time; meanwhile, it can be performed individually. “Ampe” involves a combination of good physical workouts and social bonding with critical thinking. It combines moderately high aerobic movements with intermittent active rest.

A third study explored the experiences of postmenopausal women with excess weight gain.

The results revealed that excess weight is viewed as attractive and symbolizes culturally appropriate body size. Lack of adequate physical activity and poor dietary habits increase the risk of weight gain. The results of these three studies were then collated with the literature findings on the development of culturally sensitive exercise-based programs, postmenopausal women and weight gain, and formed the basis of the questionnaire. The program was subsequently categorized under four main sections (health assessment, pre-exercise parameters, exercise dosage, barriers and facilitators). Integrating findings from the three provision studies provided strong evidence for both the face and content validity of the questionnaire. The content was derived directly from themes identified through earlier qualitative and survey-based research involving postmenopausal women, ensuring that the items reflected their lived experiences, health needs, and culturally relevant exercise behaviors. The questionnaire was reviewed by a multidisciplinary panel of experts, including physiotherapists, women’s health specialists, exercise scientists and public health researchers, who evaluated each item for relevance, clarity, and comprehensiveness in addressing the intended domains. Additionally, postmenopausal women from the target population reviewed the questionnaire, confirming that the items were clear, meaningful, and representative of their concerns. This dual process of expert appraisal and target population feedback supports the instrument’s face and content validity. In a standard Delphi survey, expert advice is sought during the questionnaire development; however, in this case, the questionnaire was developed using information from the three different studies mentioned above and literature findings. Therefore, a modified Delphi methodology was applied in this survey.

### Procedure

The respondents completed three surveys in succession, and each round was modified in response to comments from the preceding version. A special, secured “REDCap” link was used to deliver each survey to the experts. REDCap is a secured web-based application and workflow methodology designed to collect and manage data for research studies [17]. A summary of the first round’s findings was emailed to panellists who completed the first round’s survey to serve as an introduction to the second round’s survey, and a summary of the second round’s findings was sent with the third round’s survey. The summaries did not contain any personally identifiable information. Participants were requested to complete each questionnaire within three weeks. Those participants who had not answered in two weeks were sent e-mail reminders. Participants were given extra reminders and more time to respond if they were unable to finish the questionnaires within the given three weeks. For our study, 80% agreement [15] was chosen as the threshold for consensus according to the recommendations of Deane et al. [14].

### Round 1

The questionnaire was sent via email to the panellists/participants. Demographic data for the expert panel was gathered using multiple-choice questions (MCQs). The first questionnaire mostly included open-ended questions to let participants express their ideas without being given any leading information. This method reduces response bias [18]. Respondents were asked three main questions in this round. Firstly, they were asked to list and provide dosage variables they consider important when prescribing low-impact cardiovascular and strength/resistance exercises for the management of excess weight among postmenopausal women. Secondly, participants were asked to provide exercise prescription variables other than dosage that they consider important when prescribing exercises (low-impact cardiovascular and strength/resistance exercises). Finally, participants were also asked to indicate personal (postmenopausal women) and other factors they consider important that may change the exercise variables identified. Participants were also allowed to provide comments. Responses to the open-ended questions in the first-round questionnaire were analysed thematically to generate a qualitative summary of key ideas. The aim was to extract recurring concepts and suggestions that could be translated into clear, actionable statements for Round 2. Three researchers (HM, CB, AT) independently reviewed the responses, identified preliminary codes, and grouped them into thematic categories. Regular discussions were held to compare interpretations and resolve discrepancies, ensuring consistency and validity in the analysis. The final themes were agreed upon by consensus and formed the basis for developing the statements used in the second questionnaire.

### Round 2

All respondents who participated in the first round of the survey were invited to participate in the second iteration. Based on themes found in responses to the first questionnaire, the second survey was developed. A 5-point Likert response scale was used to determine the level of agreement (“strongly agree,” “agree,” “undecided”, “disagree,” and “strongly disagree). The consensus was determined when panel members strongly agreed or agreed with an item. If the percentage of agreement was less than 75% to a question [14], however, it was concluded that a consensus had not been reached. Open-ended questions were also provided to ensure participants were able to express any further thoughts or opinions.

### Round 3

In this round, any questions that did not reach consensus during the second round were removed in the ﬁnal round. Participants were asked to evaluate the results in round two and confirm the answer to the same question. Each item that achieved 80% or more of expert votes was included in the exercise program. Additional themes identified in open-ended questions in the second round were included to ensure a thorough exploration of participant opinions.

### Data Analysis

Statistical Package for the Social Sciences (SPSS) software version 26 was used to analyse the data. Descriptive statistics were used to determine the frequency of each response. To determine the status of the agreement, the frequencies of “strongly agree” and “agree” were combined [19]. Responses to open-ended questions were summarized qualitatively using thematic analysis. The number of identiﬁed themes was noted, which were used to generate questions for subsequent rounds.

## Results

### Results: Round 1

#### Participant demographics.

Of the 41 individuals who consented to participate in the study, 37 (90.2%) responded to the first round of the Delphi survey. However, only 32 participants completed all rounds of the study. Detailed demographic data were collected only from those who completed all rounds; therefore, information was not available for the nine participants who either did not respond to the first round or dropped out in subsequent rounds. Among the 32 participants who completed all rounds, 24.2% (n = 8) held bachelor’s degrees, 30.3% (n = 10) held master’s degrees (MSc or equivalent), and 42.4% (n = 14) held doctoral degrees. Twelve of the participants were international panellists, while twenty were local panel members. International experts are individuals who have gained expertise and prominence in their respective fields on a global scale. Local experts, on the other hand, are individuals who possess specialized knowledge, experience, and expertise within the Ghanaian context. Most of the participants, 72.7% (n = 24), had expertise in both academic and clinical fields in the management of weight gain (Table 1).

**Table 1:** Demographics of participants who completed all rounds.

Category	n (%)
**Highest educational qualification**	
Bachelor’s degree	8 (*24.2%)*
Master’s degree (MSc or equivalent)	10 (*30.3%)*
Doctoral degree (PhD or equivalent)	14 (*42.4%)*
**Geographical representation**	
Local (Ghanaian context) experts	20 (*62.5%)*
International experts	12 *(37.5%)*

#### Thematic analysis of the first round.

From nine different open-ended questions in round one, a total of 25 themes were identiﬁed. These themes were used to generate questions regarding weight management programs for postmenopausal women with weight gain for subsequent rounds.

### Results: Round 2

#### Development of an exercise program.

A consensus was reached on 94.2% (n = 49) of questions related to health assessment, pre-exercise parameters, exercise dosage and the physician referral form (Table 2).

**Table 2:** Consensus: Second Round of the Delphi Questionnaire (n = 37).

Item	Consensus Agreement n (%)
General questions to consider when prescribing low-impact cardio (Ampe) and resistance exercises for postmenopausal women in Ghana.	
Has a doctor ever said that you have a heart condition and recommended only medically supervised exercise?	35(94.6)
Do you have chest pain brought on by physical activity?	34(91.9)
Have you developed chest pain in the past month when you were doing physical activity?	35(94.6)
Have you, on one or more occasions, lost consciousness or fallen over as a result of dizziness?	36(97.3)
Do you have a bone or joint problem (for example, back, knee or hip) that could be aggravated by the proposed physical activity?	35(94.6)
Has a doctor ever recommended medication for your blood pressure or a heart condition?	34(91.9)
Are you aware, through your own experience or a doctor’s advice, of any other physical reason that would prohibit you from exercising without medical supervision?	36(97.3)
Indicate how you prefer the exercise program to be delivered	30(81.1)
Do you have enough time to exercise?	31(83.8)
Are you motivated to exercise?	32(86.5)
What mode of exercise counselling do you prefer?	31(83.8)
What additional support do you need to achieve your exercise program goal?	29(78.4)
Motivational strategies that will aid your exercise program	28(75.7)
What will remind you to do exercises?	29(78.4)
When heat and humidity are high, do you wear loose-fitting, porous and lightweight clothing to exercise?	27(73.0)
What time do you prefer to exercise?	30(81.1)
Do you prefer a less expensive exercise program?	31(83.8)
Which dietary self-monitory approach will enhance the efficacy of your weight management program?	30(81.1)
**Pre-exercise parameters for Low impact-cardio exercise**	
Step test will be used to measure aerobic fitness	32(86.5)
**Pre-exercise parameters for resistance exercise**	
The Senior Fitness Test (SFT) will be used to assess key physiological parameters (e.g., strength, endurance, agility, balance)	33(89.2)
**Exercise dosage variable (cardio exercise) (Ampe) Fig A in** S1 Text.	
**Number of sets**	
3 – 4 sets	34(91.9)
Frequency	
3 – 4 times/week	33(89.2)
Repetition	
10-15 reps	35(94.6)
Duration	
10-15 minutes	34(91.9)
**Resistance exercise dosage**	
**Number of sets (theraband exercise). Fig B in** S1 Text.	
4 sets	28(75.7)
Frequency	
3-5 times/ week	28(75.7)
Repetition	
10 reps	30(81.1)
Duration	
15-30 minutes	29(78.4)
**Number of sets (squat to chair exercise) Fig C in** S1 Text.	
4 sets	34(91.9)
Frequency	
3–5 times/ week	34(91.9)
Repetition	
10 reps	30(81.1)
Duration	
15–30 minutes	31(83.8)
**Number of sets (wall push-ups) Fig D in** S1 Text.	
4 sets	35(94.6)
Frequency	
3-5 times/ week	35(94.6)
Repetition	
10 reps	34(91.9)
Duration	
15-30 minutes	35(94.6)
**Number of sets (water bottle floor chest press) Fig E in** S1 Text.	
4 sets	31(83.8)
Frequency	
3-5 times/week	35(94.6)
Repetition	
10 reps	34(91.9)
Duration	
15-30 minutes	35(94.6)
**Number of sets (water bottle overhead press) Fig F in** S1 Text.	
4 sets	30(81.1)
Frequency	
3-5 times	34(91.9)
Repetition	
10 reps	33(89.2)
Duration	
15-30 minutes	36(97.3)
**Number of sets (standing water bottle biceps curl) Fig G in** S1 Text.	
4 sets	35(94.6)
Frequency	
3-5 times/week	33(89.2)
Repetition	
10 reps	35(94.6)
Duration	
15-30 minutes	36(97.3)
Physician referral form	36(97.3)
**Without consensus**	
Is the exercise program suitable or will need to change for you?	20(54.1)
Which one will minimize the impact and duration of a relapse?	22(59.5)
Do you have enough energy to exercise?	18(48.6)

#### Items without consensus.

Three items did not reach the predefined consensus threshold of 80% and were therefore excluded from the final program. These included: “Is the exercise program suitable or will need to change for you?” (54.1% agreement), “Which one will minimize the impact and duration of a relapse?” (59.5% agreement), and “Do you have enough energy to exercise?” (48.6% agreement). The relatively low levels of agreement suggest variability in expert perspectives on individual readiness, perceived barriers to participation, and the adaptability of the intervention to diverse personal or clinical circumstances.

### Results: Round 3

In round three, items where consensus was obtained in the second questionnaire were repeated in this round. Table 2 presents the detailed results of the consensus obtained for specific items. After round 3, all items listed in the questionnaire obtained consensus as shown in Table 3.

**Table 3:** Consensus: Third Round of the Delphi Questionnaire (n = 32).

*Item*	*Consensus* *Agreement n (%)*
Health assessment	
Has a doctor ever recommended that you only participate in medically supervised exercise due to a heart or other condition?	30(93.8)
Do you experience chest discomfort with physical exertion?	31(96.9)
Do you have pain in joints (back, knee, hip or other joints) that could be aggravated by physical activity?	30(93.8)
Do you have pain when performing the following physical activity?	31(96.9)
Have you on one or more occasions lost consciousness or fallen over as a result of dizziness?	30(93.8)
Do you have high blood pressure or a heart condition?	32(100)
Has a doctor ever recommended medication for your blood pressure or a heart condition?	29(90.6)
Are you aware, through your own experience or a doctor’s advice, of any other risk factors that would prohibit you from exercising without medical supervision?	28(87.5)
**Pre-exercise parameters**	
Step test will be used to measure aerobic fitness	30(93.8)
The Senior Fitness Test (SFT) will be used to assess key physiological parameters (e.g., strength, endurance, agility, balance)	31(96.9)
What is your nutritional status?	27(84.4)
What time of the day do you prefer to exercise?	28(87.5)
Knowing your maximum heart rate can help determine exercise intensity.	27(84.4)
Ensure you wear loose-fitting clothes to perform the following activities.	26(81.3)
Remain hydrated during the period of the exercise programme (It is suggested that drinking 2.7 litres of water per day is good for the body).	31(96.9)
What is your physical activity status?	30(93.8)
Which of the following means will serve as a preferred reminder for you to engage in exercises?	29(90.6)
Do you prefer a locally designed exercise programme that involves a combination of good physical workout and, social bonding and combines moderate to high-intensity aerobic movements with intermittent active rest patterns that can aid in weight reduction?	30(93.8)
What will motivate you to adhere to an exercise program?	28(87.5)
Indicate the mode of exercise programme delivery	26(81.3)
What support do you need to achieve your exercise program goal?	30(93.8)
How would you prefer to receive support and advice when you are exercising?	29(90.6)
**Exercise dosage**	
Moderate-intensity Ampe exercise of 3–4 sets, 3–4 times/week and 10-15 repetitions for 10–15 minutes (Ampe exercise dosage). Fig A in S1 Text.	31(96.9)
4 sets, 3–5 times/ week and 10 repetitions for 15–30 minutes (theraband dosage). Fig B in S1 Text.	28(87.5)
3 sets, 3–5 times/ week and 10 repetitions for 5–10 minutes (squat to chair). Fig C in S1 Text.	30(93.8)
3 sets, 3–5 times/week of 10 repetitions for 5–10 minutes (Wall Push-ups) Fig D in S1 Text.	31(96.9)
4 sets, 3–5times/ week of 10 repetitions for 5–10 minutes (Water-Bottle-floor Chest Press). Fig E in S1 Text.	30(93.8)
4 sets, 3–5times/ week of 10 repetitions for 5–10 minutes (Water-bottle-overhead press). Fig F in S1 Text.	31(96.9)
4 sets, 3–5times/ week of 10 repetitions for 5–10 minutes (Standing water bottle biceps curls). Fig G in S1 Text.	29(90.6)
Physician Referral Form	30(93.8)

### Discussion

Weight gain is prevalent in women at the time of menopause and is associated with the risk of coronary heart disease observed during this period [4]. Generally, menopausal women with weight gain are given generic lifestyle counselling like “exercise regularly” without consensus on the content of the exercise [20]. Therefore, there is a need for evidence-based consensus where there is an agreement on the culturally acceptable exercise program for Ghanaian women.

In this Delphi survey, 32 (94.1%) participants reached a consensus on most items relating to the content of exercise programs for weight loss among postmenopausal women with weight gain. After three rounds of questionnaires, all the items presented in the Delphi questionnaire achieved over 80% consensus. Our Delphi study helps to develop a consensus about what should be included in an exercise program for weight loss in postmenopausal women. This proposed exercise program is appropriate for Ghanaian women and could be adapted to other sub-Saharan African countries with similar acculturation. Exercise has been developed to improve the health of postmenopausal women [21,22]. To our knowledge, this is the first study to utilize and report a modified Delphi method that incorporates an indigenous physical activity, Ampe (Fig A in S1 Text), in the development of an exercise program for weight loss among postmenopausal women. By integrating Ampe*,* a culturally relevant game, into the intervention, this study contributes a novel and context-specific approach to postmenopausal care in Ghana, thereby enhancing both its relevance and potential impact. Furthermore, Ranjan et al. [23] underscore the need for evidence-based clinical practices to standardize the management of excess weight in women across diverse settings, reinforcing the importance of culturally tailored interventions such as the current study.

Participants agreed on effective exercise dosages (Fig A in S1 Text, Fig B in S1 Text, Fig C in S1 Text, Fig D in S1 Text, Fig E in S1 Text, Fig F in S1 Text, Fig G in S1 Text) for adults that are similar to other exercise recommendations for weight loss. In our study, participants agreed to include moderate-intensity aerobic “Ampe” and resistance exercises. For example, the American College of Sports Medicine recommended a minimum of 150 min/per week of moderate-intensity exercise interventions for adult weight loss [24]. They also recommend resistance exercises to increase strength and function [24].

Conducting an appropriate health assessment and pre-exercise parameters before an exercise program can play a crucial role in identifying specific barriers [10,25]. Barriers, including body image issues, joint pain, heart-related conditions, lack of time, lack of social support, and household and work-related responsibilities, need to be addressed for appropriate weight loss [10,25]. For example, the health assessment can reveal an unexpected condition or add to the information already collected [26].

The Delphi technique provides for the organizing of group communication to identify a collective consensus of professional opinion on an issue [27]. The Delphi participants in our study were informed of the findings, which may have influenced their decision-making regarding the exercise program’s content. A study by Williams et al., [28] affirmed this conclusion that the first time a rating is presented, an independent opinion can be accurate; however, afterwards, the respondent may be potentially influenced by the group findings. Therefore, researchers must exercise caution in the understanding of consensus. This possible influence raises questions about the study’s validity.

The obtained response rate is essential to the Delphi method’s validity. The response rate in our study was higher compared to previous studies [29,30]. Shaw et al. [29] reported a 66% response rate while Sowell et al. [30] reported a 79% response rate in the second round. Neither of the two investigations had response rates that were stronger than our study.

The lack of discussion of the themes is one disadvantage of employing the Delphi technique that is noted in the literature [31]. However, the development of the questionnaire in our study explored weight management practices of postmenopausal women with excess weight. This provided an in-depth understanding of the physical activity and lifestyle levels of postmenopausal women.

The findings of this study have meaningful implications for other low- and middle-income countries (LMICs), particularly within sub-Saharan Africa, where cultural norms, limited infrastructure, and economic constraints similarly influence health behaviors and access to care [32]. The use of culturally embedded activities such as “Ampe” reflects a pragmatic, context-sensitive approach to physical activity promotion that may enhance acceptance and sustainability in comparable settings [33,34]. Grounding health interventions in culturally relevant practices has been shown to improve engagement, adherence, and outcomes in LMIC populations [35]. Moreover, the consensus-derived statements from this Delphi study offer a potential framework for adapting weight loss interventions in other African countries with similar sociocultural dynamics. These findings underscore the importance of co-developing public health interventions that align with local realities, traditions, and constraints to improve health outcomes at scale [33]. Future research should examine the transferability and effectiveness of such culturally grounded interventions across diverse sub-Saharan African settings.

Throughout the Delphi process, several factors influenced experts’ participation. The common barriers, such as time constraints and competing professional responsibilities, could have affected participation. These were particularly evident among participants from clinical settings with limited availability. However, key facilitators that supported sustained participation included strong professional interest in the research topic, cultural relevance of the exercise program. Additionally, the perceived value of contributing to a locally adapted health intervention may be a motivating factor.

## Limitations

While the Delphi method used in this study followed a structured and transparent process, several limitations should be acknowledged. First, our approach used data from three prior surveys instead of a traditional iterative approach to determine the program content. The list of items derived from these earlier studies served as a foundational reference in the first round. Although this provided a strong evidence-based foundation, it may have introduced selection bias by pre-framing the scope of items considered, potentially limiting the development of novel ideas. Second, the inclusion of the indigenous Ghanaian physical activity “Ampe” presented both a strength and a challenge. Despite providing detailed background information to all participants, international panellists were less familiar with the activity, which may have influenced their evaluations. This underscores the need to promote and validate culturally specific physical activities in global health discourse, potentially even advocating for broader platforms such as the Olympic Games. Lastly, because the intervention incorporated Ghanaian household activities, the generalizability of the findings may be limited to similar cultural or contextual settings. Nonetheless, the methodological approach and rationale provide a useful framework that can be adapted to develop culturally responsive interventions in other regions.

## Conclusion

The strong agreement in the findings provides a framework for exercise professionals and healthcare teams to incorporate exercise programs into the health management of postmenopausal women with excess weight. Building on the strong consensus achieved in this study, future research should focus on the implementation and rigorous evaluation of the proposed culturally adapted exercise program in real-world clinical and community settings. Specifically, randomized controlled trials (RCTs) are warranted to assess the effectiveness, adherence, and long-term health outcomes of the intervention among postmenopausal women with excess weight.

## Supporting information

S1 Text Fig A in S1 Text, Fig B in S1 Text, Fig C in S1 Text, Fig D in S1 Text, Fig E in S1 Text, Fig F in S1 Text, Fig G in S1 Text, and exercise dosages for the program. (DOCX)

## References

[1] Liu JH. Multimorbidity in postmenopausal women: a new health challenge. Menopause. 2024;31(11):943–4. doi: 10.1097/GME.0000000000002445 39465993

[2] Abildgaard J, Ploug T, Al-Saoudi E, Wagner T, Thomsen C, Ewertsen C, et al. Changes in abdominal subcutaneous adipose tissue phenotype following menopause is associated with increased visceral fat mass. Scientific reports, 2021;11(1):14750. 34285301 10.1038/s41598-021-94189-2 PMC8292317

[3] Abildgaard J, Danielsen ER, Dorph E, Thomsen C, Juul A, Ewertsen C, et al. Ectopic Lipid Deposition Is Associated With Insulin Resistance in Postmenopausal Women. J Clin Endocrinol Metab. 2018;103(9):3394–404. doi: 10.1210/jc.2018-00554 29889238

[4] Kapoor E, Collazo-Clavell ML, Faubion SS. Weight gain in women at midlife: a concise review of the pathophysiology and strategies for management. Mayo Clinic Proceedings. Elsevier. 2017;1552–8. 10.1016/j.mayocp.2017.08.004 28982486

[5] Durrer Schutz D, Busetto L, Dicker D, Farpour-Lambert N, Pryke R, Toplak H, et al. European Practical and Patient-Centred Guidelines for Adult Obesity Management in Primary Care. Obes Facts. 2019;12(1):40–66. doi: 10.1159/000496183 30673677 PMC6465693

[6] Deledda A, Annunziata G, Tenore GC, Palmas V, Manzin A, Velluzzi F. Diet-Derived Antioxidants and Their Role in Inflammation, Obesity and Gut Microbiota Modulation. Antioxidants (Basel). 2021;10(5):708. doi: 10.3390/antiox10050708 33946864 PMC8146040

[7] Pisanu S, Palmas V, Madau V, Casula E, Deledda A, Cusano R, et al. Impact of a Moderately Hypocaloric Mediterranean Diet on the Gut Microbiota Composition of Italian Obese Patients. Nutrients. 2020;12(9):2707. doi: 10.3390/nu12092707 32899756 PMC7551852

[8] Sekome K, Gómez-Olivé FX, Sherar LB, Esliger DW, Myezwa H. Sociocultural perceptions of physical activity and dietary habits for hypertension control: voices from adults in a rural sub-district of South Africa. BMC Public Health. 2024;24(1):2194. doi: 10.1186/s12889-024-19320-0 39138450 PMC11320885

[9] Bucciarelli V, Bianco F, Mucedola F, Di Blasio A, Izzicupo P, Tuosto D, et al. Effect of Adherence to Physical Exercise on Cardiometabolic Profile in Postmenopausal Women. Int J Environ Res Public Health. 2021;18(2):656. doi: 10.3390/ijerph18020656 33466649 PMC7828719

[10] Chopra S, Malhotra A, Ranjan P, Vikram NK, Sarkar S, Siddhu A, et al. Predictors of successful weight loss outcomes amongst individuals with obesity undergoing lifestyle interventions: A systematic review. Obes Rev. 2021;22(3):e13148. doi: 10.1111/obr.13148 33200547

[11] Obi OC, Nnonyelu AC, Onobrakpeya A, Ogundele OJ. Benefits and barriers to physical activity among African women: A systematic review. Sports Med Health Sci. 2022;5(1):59–66. doi: 10.1016/j.smhs.2022.12.001 36994171 PMC10040374

[12] Small C. Developing culturally sensitive guidelines to address obesity in black women. Walden University. 2014.

[13] Nasa P, Jain R, Juneja D. Delphi methodology in healthcare research: How to decide its appropriateness. World J Methodol. 2021;11(4):116–29. doi: 10.5662/wjm.v11.i4.116 34322364 PMC8299905

[14] Deane KHO, Ellis-Hill C, Dekker K, Davies P, Clarke CE. A Delphi Survey of Best Practice Occupational Therapy for Parkinson’s Disease in the United Kingdom. British Journal of Occupational Therapy. 2003;66(6):247–54. doi: 10.1177/030802260306600603

[15] Alder M, Ziglio E. Gazing into the oracle: The Delphi method and its application to social policy and public health. London: Jessica Kingsley. 1996.

[16] MOmoniyi MM, Afrifa D, Asamoah MA, Sarpong P, Sarpong E, Appiah PO, et al. “AMPE” Exercise Programme Has Positive Effects on Anthropometric and Physiological Parameters of School Children: A Pilot Study. Ethiop J Health Sci. 2020;30(1):143–6. doi: 10.4314/ejhs.v30i1.18 32116443 PMC7036453

[17] Harris PA, Taylor R, Thielke R, Payne J, Gonzalez N, Conde JG. Research electronic data capture (REDCap)--a metadata-driven methodology and workflow process for providing translational research informatics support. J Biomed Inform. 2009;42(2):377–81. doi: 10.1016/j.jbi.2008.08.010 18929686 PMC2700030

[18] Choi BCK, Pak AWP. A catalog of biases in questionnaires. Prev Chronic Dis. 2005;2(1):A13. 15670466 PMC1323316

[19] Naidoo V, Stewart AV, Maleka MED. A tool to evaluate physiotherapy clinical education in South Africa. S Afr J Physiother. 2022;78(1):1759. doi: 10.4102/sajp.v78i1.1759 36092966 PMC9453145

[20] Khandelwal S. Obesity in midlife: lifestyle and dietary strategies. Climacteric. 2020;23(2):140–7. doi: 10.1080/13697137.2019.1660638 31576760

[21] Asikainen T-M, Kukkonen-Harjula K, Miilunpalo S. Exercise for health for early postmenopausal women: a systematic review of randomised controlled trials. Sports Med. 2004;34(11):753–78. doi: 10.2165/00007256-200434110-00004 15456348

[22] Velthuis MJ, Schuit AJ, Peeters PHM, Monninkhof EM. Exercise program affects body composition but not weight in postmenopausal women. Menopause. 2009;16(4):777–84. doi: 10.1097/gme.0b013e318197122a 19188848

[23] Ranjan P, Vikram NK, Choranur A, Pradeep Y, Ahuja M, Puri M, et al. Executive summary of evidence and consensus-based Clinical Practice Guidelines for management of obesity and overweight in midlife women: An AIIMS-DST initiative. Diabetes Metab Syndr. 2022;16(3):102426. doi: 10.1016/j.dsx.2022.102426 35248973

[24] Donnelly JE, Blair SN, Jakicic JM, Manore MM, Rankin JW, Smith BK, et al. American College of Sports Medicine Position Stand. Appropriate physical activity intervention strategies for weight loss and prevention of weight regain for adults. Med Sci Sports Exerc. 2009;41(2):459–71. doi: 10.1249/MSS.0b013e3181949333 19127177

[25] Chiu H-H, Wu S-F, Wang P-H. The experience of menopausal women participating in weight management program: A pilot study. Taiwan J Obstet Gynecol. 2020;59(5):686–90. doi: 10.1016/j.tjog.2020.07.011 32917319

[26] Dudley-Brown S. The importance of the physical examination. Gastroenterol Nurs. 2012;35(5):350–2. doi: 10.1097/SGA.0b013e31826b16b8 23018171

[27] Slade SC, Dionne CE, Underwood M, Buchbinder R. Standardised method for reporting exercise programmes: protocol for a modified Delphi study. BMJ Open. 2014;4(12):e006682. doi: 10.1136/bmjopen-2014-006682 25550297 PMC4281530

[28] Williams JK, Tripp-Reimer T, Schutte D, Barnette JJ. Advancing genetic nursing knowledge. Nurs Outlook. 2004;52(2):73–9. doi: 10.1016/j.outlook.2003.10.013 15073586

[29] Shaw LR, Leahy MJ, Chan F, Catalano D. Contemporary Issues Facing Rehabilitation Counseling: A Delphi Study of the Perspectives of Leaders of the Discipline. Rehab Educ. 2006;20(3):163–78. doi: 10.1891/088970106805074430

[30] Sowell RL. Identifying HIV/AIDS research priorities for the next millennium: a Delphi study with nurses in AIDS care. J Assoc Nurses AIDS Care. 2000;11(3):42–52. doi: 10.1016/S1055-3290(06)60275-6 10826303

[31] Philips B Jr, Anderson G, Ridl K. Establishing a women’s health curriculum using the Delphi Method. Educ Health (Abingdon). 2003;16(2):155–62. doi: 10.1080/1357628031000116934 14741901

[32] Ebrahim S, Pearce N, Smeeth L, Casas JP, Jaffar S, Piot P. Tackling non-communicable diseases in low- and middle-income countries: is the evidence from high-income countries all we need?. PLoS Med. 2013;10(1):e1001377. doi: 10.1371/journal.pmed.1001377 23382655 PMC3558465

[33] Giles AR, Darroch FE. The need for culturally safe physical activity promotion and programs. Can J Public Health. 2014;105(4):e317-9. doi: 10.17269/cjph.105.4439 25166136 PMC6972485

[34] Soto S, Chavez A, Haughton J, Arredondo EM. Development of Culturally Appropriate Strategies for Promoting Physical Activity. Physical Activity in Diverse Populations. Routledge. 2017. 26–44. doi: 10.4324/9781315561264-3

[35] Ehrlich C, Kendall E, Parekh S, Walters C. The impact of culturally responsive self-management interventions on health outcomes for minority populations: A systematic review. Chronic Illn. 2016;12(1):41–57. doi: 10.1177/1742395315587764 26026156

