# Supplementary material for: Culturally adapted exercise program for postmenopausal women with excess weight gain: A modified international/local Delphi study
Source: PLOS Glob Public Health. 2025 Sep 11;5(9):e0005083. doi: 10.1371/journal.pgph.0005083 (PMC12425256; doi:10.1371/journal.pgph.0005083)
Supplement: S1 Text — (DOCX) [file pgph.0005083.s001.docx]

**Ghanaian indigenous physical activity (Ampe) exercise dosage**

**
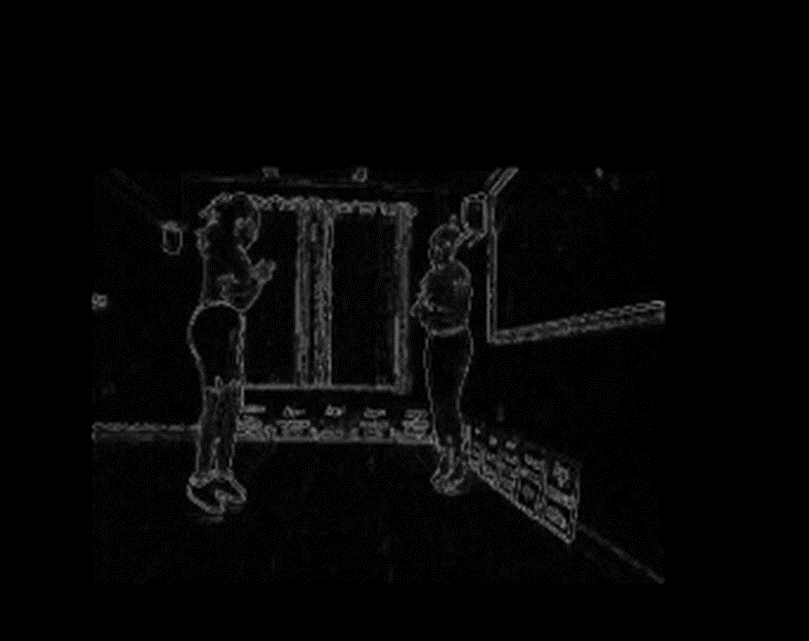
**

**Fig A.** Photograph showing Ampe exercise with two individuals in jumping, ascending and descending movement.

Exercise Dosage: Moderate-intensity Ampe exercise of 3-4 sets, 3-4 times/week and 10-15 repetitions for 10-15 minutes

**Theraband exercise (Resistance exercise)**


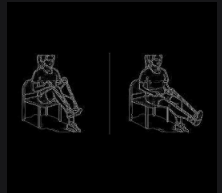


**Fig B:** Photograph showing exercise set-up with the patient seated in a chair with hand support, holding the theraband and performing exercise by extension and flexion of the elbow and knee

Exercise Dosage: 4 sets, 3-5 times/ week and 10 repetitions for 15-30 minutes

**Squat to chair exercise (Resistance exercise)**

**
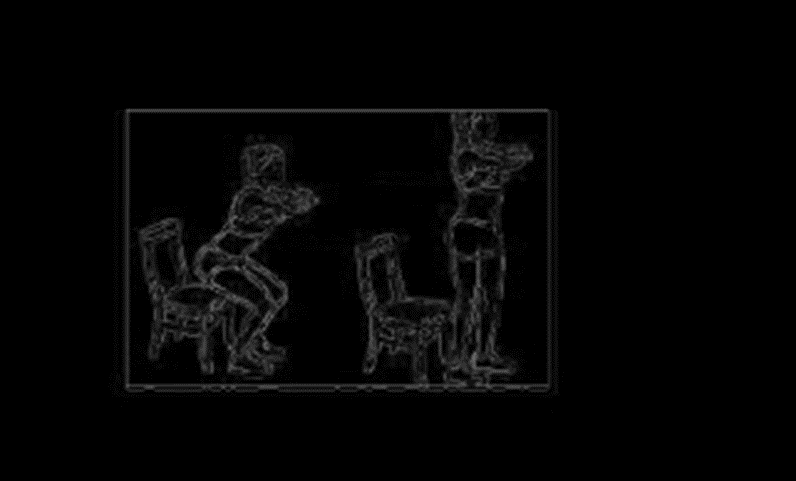
**

**Fig C**: Photograph showing exercise set-up with the patient performing exercise by sitting and standing

Exercise Dosage: 3 sets, 3-5 times/ week and 10 repetitions for 5-10 minutes

**Wall Push-ups (Resistance exercise)**

**
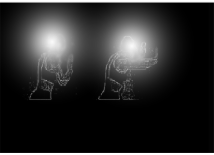
**

**Fig D:** Photograph showing exercise set-up with the individual pushing the wall with the forearm and performing the exercise by extension and flexion of the elbow

Exercise Dosage: 3 sets, 3- 5 times/week of 10 repetitions for 5-10 minutes

**The Water Bottle Exercise (Resistance exercise)**

**Water-Bottle-floor Chest Press**


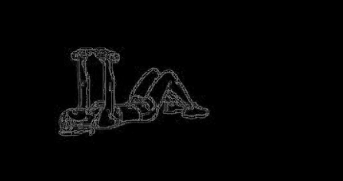


**Fig E.** Photograph showing exercise set-up with an individual lying on their back and holding a plastic container with a specified amount of water, and performing exercise by lifting and lowering the container by extension or flexion of the elbow joint

Exercise Dosage: 4 sets, 3-5times / week of 10 repetitions for 5-10 minutes

**Water-bottle-overhead press**

**
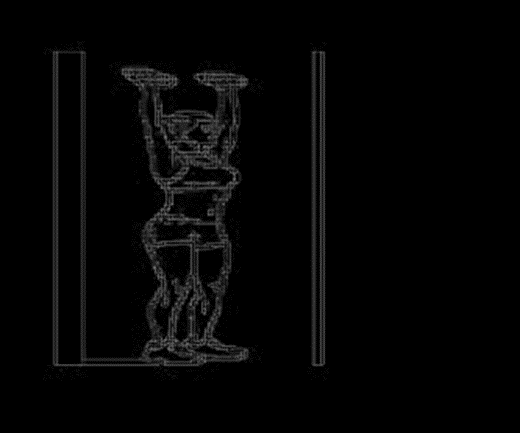
**

**Fig F**: Photograph showing exercise set-up with an individual standing and holding a plastic container with a specified amount of water and performing exercise by lifting and lowering the container by extension and flexion of the shoulder joint

Exercise Dosage: 4 sets, 3-5times / week of 10 repetitions for 5-10 minutes

**Standing water bottle biceps curls**


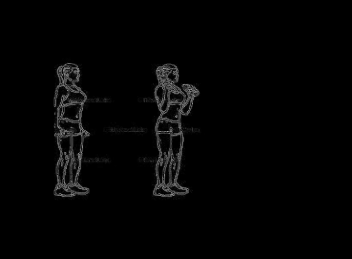


**Fig G.**: Photograph showing exercise set-up with an individual standing and holding a plastic container with a specified amount of water and performing exercise by lifting and lowering the container by extension and flexion of the elbow joint

Exercise Dosage: 4 sets, 3-5times / week of 10 repetitions for 5-10 minutes
